# Comparison of the developmental potential of 2-week-old preantral follicles derived from vitrified ovarian tissue slices, vitrified whole ovaries and vitrified/transplanted newborn mouse ovaries using the metal surface method

**DOI:** 10.1186/1472-6750-8-38

**Published:** 2008-04-04

**Authors:** Ta-Chin Lin, Jui-Mei Yen, Tsung-Cheng Kuo, Kun-Bing Gong, Kung-Hao Hsu, Teng-Tsao Hsu

**Affiliations:** 1 Departments of Gynecology, Obstetrics and infertility, Kuo General Hospital, No.22, section 2, Ming – Sheng Road, Tainan, 70054, Taiwan; 2 Department of Pediatrics, Sinlau Christian Hospital, No.57, Section1, East-gate Road, Tainan 701, Taiwan; 3 Department of Research, Eupo Biotechnology Co., No 56.5F-2 Section 2, Ming Sheng Road, Tainan, 70054, Taiwan; 4 Department of animal science and biotechnology, Tunghai university. Taichung, 407, Taiwan

## Abstract

**Background:**

Cryopreservation of preantral follicles or ovarian tissues would enable the storage of large numbers of primordial follicles or preantral follicles and preserves the structural integrity of somatic and reproductive cells. In the present study, we compared the developmental potential of cryopreserved two-week-old mouse preantral follicles, ovarian tissue slices, two-week-old mouse ovaries and newborn mouse ovaries using a metal plate with a high cooling rate for cooling the droplet of vitrification solution.

**Methods:**

Groups of 2 to 4 samples (including of 14-day old preantral follicles, ovarian tissue slices, whole ovaries, and whole newborn ovaries) were exposed to 4% ethylene glycol (EG) in DPBS + 10% FBS for 15 min and then rinsed in a vitrification solution composed of 6 M ethylene glycol and 0.4 M trehalose in DPBS + 10% FBS. Equilibration in room temperature was performed for 20–30 seconds for preantral follicle and 5 min equilibration was performed in an ice bath for ovaries. The samples were dropped onto the surface of metal plate around -180°C in the volume of 2 μl and 6 μl. After thawing, the ovarian tissue was mechanically isolated for collecting the preantral follicles. The thawed newborn ovaries were transplanted under the renal capsule of recipient male mice for 14 days. Preantral follicles collected from each groups were cultured individually in 20-μl droplets of α-MEM culture medium in culture dish for 12 days. On the day 12 of culture, the cumulus-oocyte complexes (COCs) were collected for IVM and IVF. Fertilization and embryo cleavage were scored.

**Results:**

After the vitrification of 14-day-old preantral follicles using 2 μl or 6 μl droplet onto surface of metal plate, the results indicated that no significant difference in survival rate, antral-like cavity formation, COCs collected, 2 cell embryo cleavage and blastocyst development was found in vitrification of the 2 μl and 6 μl droplet groups. As comparing 14-day old ovarian tissue (ovarian tissue slices and whole ovaries) and whole newborn ovaries vitrified in 6 μl droplet, lower success rates of antral-like cavity formation and COCs collection were found in the whole ovaries group.

**Conclusion:**

Our results suggest that the metal plate surface vitrification method is an appropriate and convenient method for cryopreservation of mouse ovaries and preantral follicles. The droplet volume of vitrification solution in 2 μl and 6 μl can be an option.

## Background

The mammalian ovary at birth contains a large store of follicles of which only a small number will be used during the reproductive lifespan of the female. In the recent decades, important advances have been made in the development of techniques for rescue and in vitro growth of primordial follicles and preantral follicles.

Cryopreservation of preantral follicle or ovarian tissues enables the storage of the large numbers of primordial follicles or preantral follicles and preserves the structural integrity of somatic and reproductive cells. Recently, some promising results (live offspring birth) have been obtained using vitrified mouse ovary or preantral follicles [1-3] Nevertheless, further simplification of the process of vitrification for large volume of ovary or large numbers of preantral follicles for methological improvements is needed to make it less labor intensive and to improve the success rates.

Previous reports described the successful use of cryopreserved human ovarian tissues to obtained live birth [4,5]. This technique is primarily used to restore fertility resulting from a medical treatment, disease process or even natural loss from aging. Animals (mouse and ewe) have been used as experimental models to set up the experimental protocols for freezing, grafting and culture systems because of ease in handling and similarity to the human ovary. Moreover, to preserve animal genetic diversity, notably for the conservation of endangered species, or to avoid the risk of inbreeding in domestic animal, cryopreservation of ovarian tissue could present a means for enlarging the gene pool [6].

Cryoperservation of metaphase II oocytes gives disappointing results because of problems encountered during fertilization and embryonic development [7-13]. The freezing of germinal vesicle oocytes presents no risk of aneupoidy but hardening of the zona and damage to the cytoskeleton is still observed [14]. More recent research has focused on the cryopreservation of immature oocytes contained in primordial follicles, which represents the definitive number of female gametes for the entire reproductive life span. The survival rates of cryopreserved primordiol follicles are high [1-3]. Histological studies suggested that the cryopreservation was an efficient method for storage of immature oocytes within ovary and did not cause harmful damage to follicular cells and oocytes of primary follicles with intact cell organelles and nucleus except some swelling mictochondria with partial cristae disappearing and fewer cortical granule in cortex of oocyte under electron microscopy [15-18]. Recently, culture systems for preantral follicles [19,20] and primordial follicles isolated from newborn mice ovary [21] have also been successfully established and used to obtain live offspring. Nevertheless, cryopreservation appears to induce the expression of stress proteins, DNA damages to genes and to suppress the expression of some receptors and protein kinases [22].

Cryopreservation of primordial follicles or ovarian tissues of young mice with the slow freezing method [3,23] and the vitrification method have been reported [2]. A cryotube [1,23] or 0.25 ml French straw [2] was used as a conventional method for freezing. The achievable cooling rate by direct plunging into liquid nitrogen was rather compromised (2500°C/min) [24,25]. However, the survival rates of vitrified embryos plunged with the cryotube or straw into liquid nitrogen decreased dramatically when the volume of the cell was larger than the size of one blastomere of eight-cell stage embryos [26-29]. New methods based on the immersion of very small amounts of vitrification solution into liquid nitrogen have now been developed and most authors have obtained improved developmental rates of larger cells after thawing. These vitrification methods include droplet plunging directly into liquid nitrogen [30-32], electron microscope grids [33], nylon loop [34], liquid nitrogen slush [27,28], open pulled straw technology [35], droplet on a metal cube surface [36,37]. The cooling rate in this method is approximately 20000–40000°C/min. Among these methods, the droplet in metal cube surface method seems the most suitable for vitrification for ovary.

The objective of this study was tried to establish a feasible method for cryopreservation of mouse preantral follicles and ovaries. The droplet on the surface of metal plate vitrification method was used. We compared development potential of vitrified preantal follicles and preantral follicles isolated from vitrified ovarian tissue slices, from whole ovaries of 2-week-old mice, or from vitrified-thawed/transplanted ovaries of new born mice. We also compared the survival rates of preantral follicles and ovaries vitrified in 2 μl and 6 μl droplet directly plunged down on metalplate surface. The preantral follicles obtained were cultured for 12 days in droplet of culture plate.

## Results

### Survival rates of preantral follicles derived by isolation from fresh ovaries of two-week-old mice after vitrification-thawing. (follicle survival of Fig. 1)

**Figure 1:**
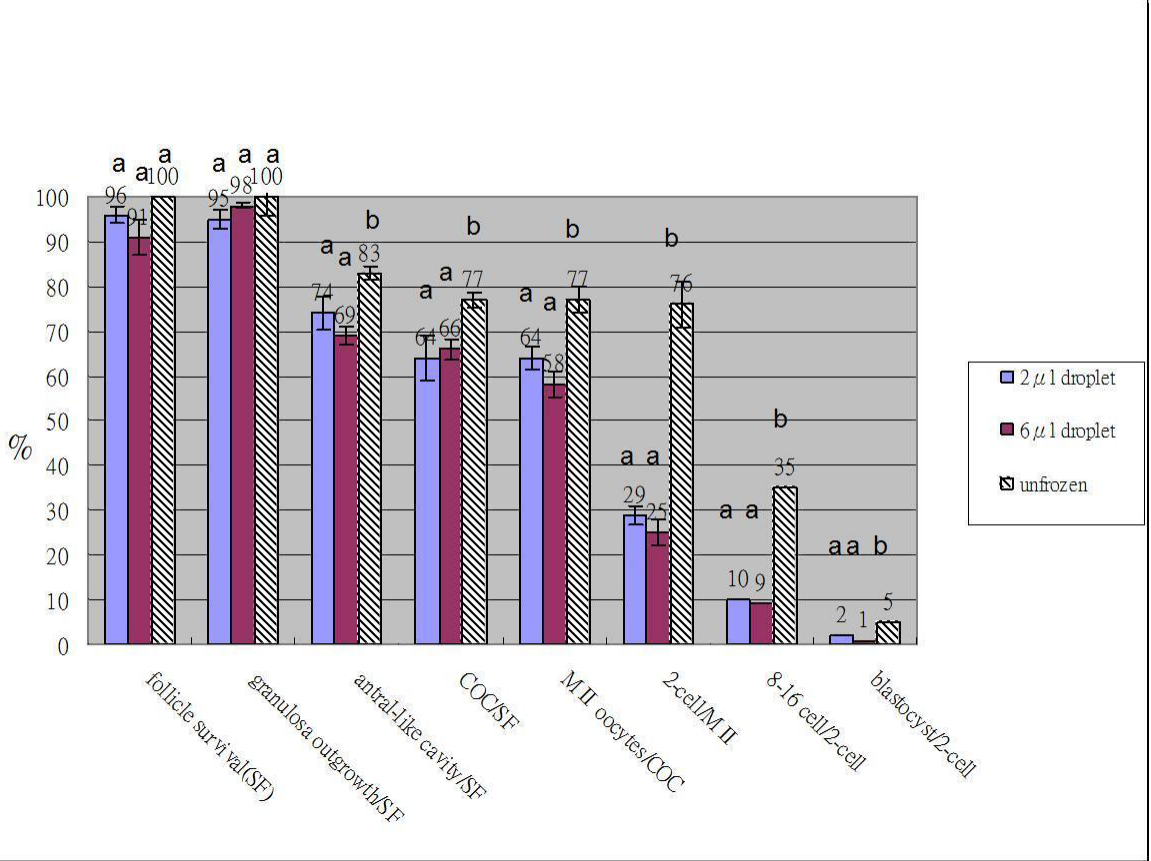
**The survival percentage and developmental competence of vitrified-thawed preantal follicles**. Comparison of the survival percentage and developmental competence of preantal follicles frozen-thawed by using the vitrification method of dropping on the surface of a metal plate vitrification method in 2 μl droplets (n = 115), 6 μl droplets (n = 114), and unfrozen control group (n = 127). The preantral follicles were derived by isolation from 14-day-old mouse ovaries. Means with different letters for each end points were significantly different (P < 0.05).

High survival rates of vitrified-thawed preantral follicles isolated from two-week-old mice ovaries were obtained regardless of the treatments used (96% and 91% respectively, Fig. 1). There were no significant differences in the survival rates between preantral follicles vitrified in 2 μl or 6 μl droplet. The survival rates were evaluated under an inverted microscope equipped with a Hoffman contrast modulation system on the day after thawing and on day 3 of culture. Survival was defined as follicles that retained their oocytes within the granulosa cell mass and was expressed as a percentage of follicles placed in the culture. The phenomena of collapse of a morphological normality of preantral follicles, darkness of follicles, zona pellucida disappearance, oocyte cytoplasma shrinkage, granulosa cell morphological abnormality, cracked basal membrane indicated that preantral follicles were damaged during freezing and thawing.

### Survival rates of preantral follicles isolated from vitrified-thawed ovarian tissue slices, whole ovaries derived from two-week-mice, and vitrified/thawed/transplanted newborn mice ovaries (follicle survival of Fig. 2)

**Figure 2:**
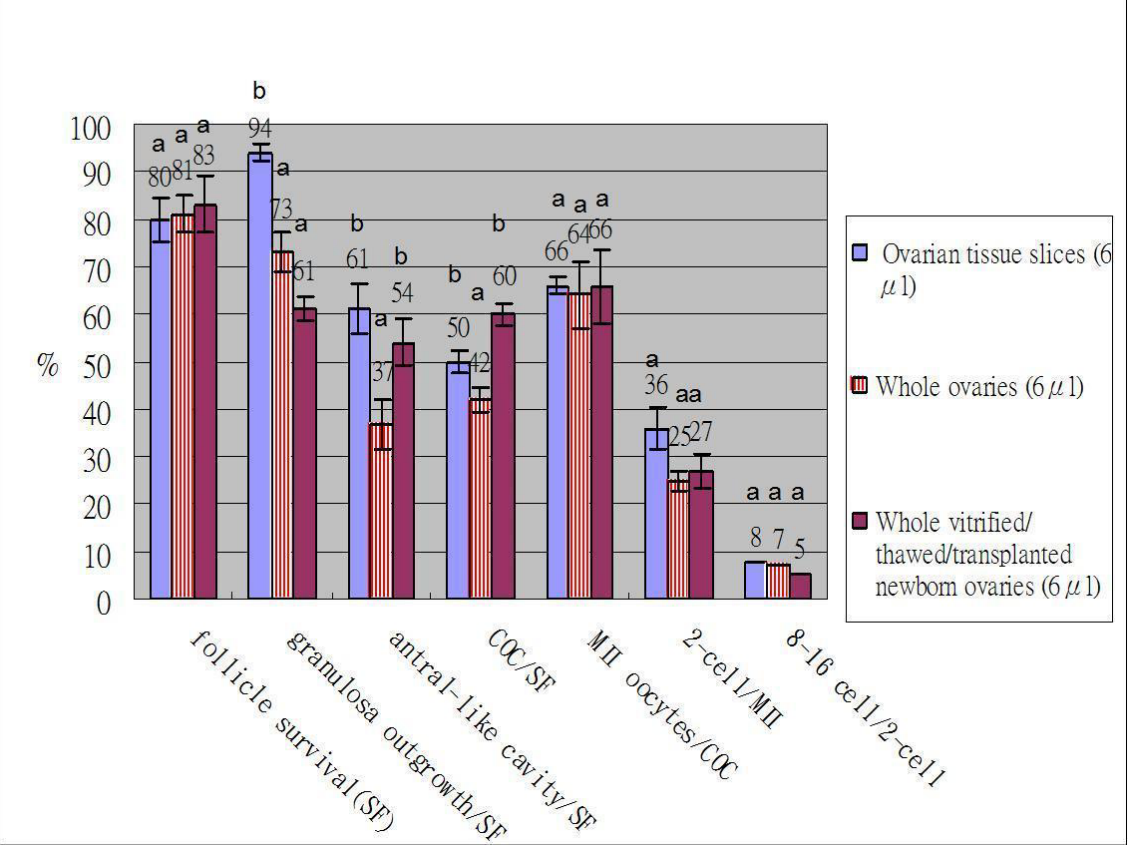
**Comparison of cultured preantal follicles derived from vitrified-thawed ovarian tissue and whole varies**. Comparison of the survival percentage and developmental competence of preantal follicles derived from vitrified-thawed ovarian tissue slices (n = 112), whole ovaries (n = 94) and transplanted whole newborn mice ovaries (n = 108) using the metal plate vitrification method with 6 μl droplet. Means with different letters for end points were significantly different (P < 0.05).

As shown in Fig. 2, there were no differences in the survival rates of preantral follicles derived from vitrified-thawed ovarian tissue slices, whole ovaries of 2-week-old mice, and ovaries of vitrified/thawed/transplanted newborn mice (80%, 81% and 83% respectively) when the 6 μl droplet in the metalplate surface vitrification method was used. It was not easy to perform a task in the 2 μl droplet vitrification because the volume of ovary was too large to pass the opening of the small tip. Nevertheless, the promising results were obtained using 6 μl droplet.

### Morphological observations on growth, differentiation, and survival of preantral follicles derived from vitrified preantral follicles, vitrified ovaries, and vitrified newborn mice ovaries

**Figure 3:**
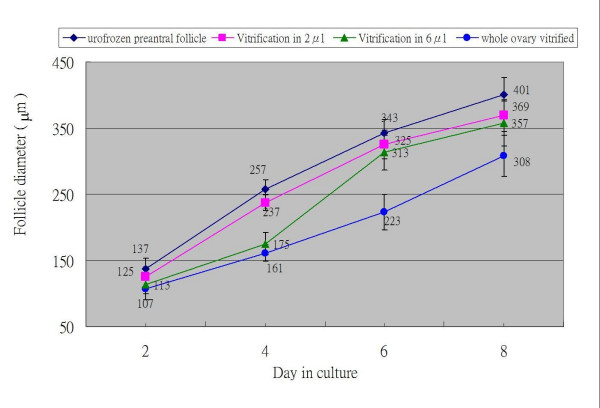
**Growth rate of 14-day old preantral follicles derived from vitrified mouse preantral follicles and vitrified whole ovaries**. The granulosa cells outgrowth rate of 14-day old preantral follicles (diamond, unfrozen preantral follicle, n = 40; square, vitrification in 2 μl volume, n = 40; triangle, vitrification in 6 μl volume, n = 38), and 14-day old preantral follicles derived from frozen-thawed transplanted ovaries of newborn mice (circle, vitrified in 6 μl volume, n = 31). No significant difference was found in the diameter of the granulosa cell outgrowth of follicles after 8 day of culture between in 2 μl and 6 μl vitrification groups. The comparison of granulosa outgrowth was not demonstrated at day 10 and day 12, because the overgrowth of the antral cavity and hatched oocyte from antral cavity were frequently found in preantral follicles from 14-day old mice.

**Figure 4:**
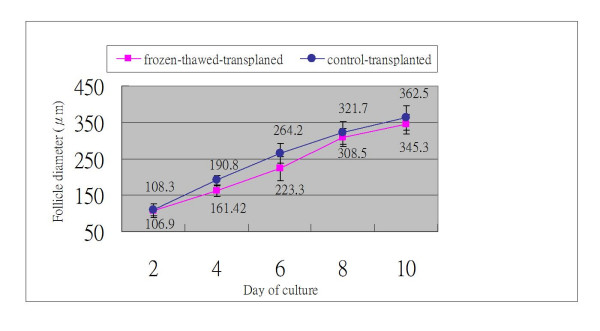
**Growth rate of preantral follicles derived from frozen-thawed and transplanted newborn mouse ovary**. The granulosa cells outgrowth rate of preantral follicle after isolation from fresh transplanted ovaries (circle, n = 28) and frozen-thawed transplanted ovaries (square, n = 31) during culture for 10 days in IVG medium. These ovaries were collected from newborn mice and immediately frozen-thawed with the vitrification method and then transplanted to the renal capsule of recipient mice for 14 days. The fresh ovaries were collected from newborn mice and only transplanted without freezing. No significant difference was found in the diameter of the granulosa cell outgrowth of follicles after 10 day of culture.

Almost all surviving vitrified-thawed preantral follicles developed to a granulosa cell outgrowth pattern after 12 days of culture. There was no significant difference (95%, 98% and 100%) of granulosa cell outgrowth between the 2 μl droplet, 6 μl droplet vitrification of 2-week-old preantral follicles, and fresh control group (Fig. 1). The granulosa cell outgrowth pattern was characterized by spindle-shaped cells originating from the surface of the follicle attaching themselves to the dish and proliferating. The granulosa cells proliferated and broke through the basal membrane, spreading over the basal membrane and the already formed monolayer. The initial follicular structure was lost and the follicles developed a diffuse appearance. The diameter of the basal membrane and granulosa cell outgrowth of preantral follicles was measured every other day during the culture period. A faster growth rate was observed with 2 μl droplet vitrification than 6 μl droplet vitrification of 2-week-old preantral follicles (Fig. 3), however, no difference was found in the diameter of the granulosa cell outgrowth of follicles on the last day culture. There was the same phenomonom observed in the frozen-thawed-transplanted newborn mice ovaries with 6 μl droplet vitrification method and then transplanted to the renal capsule of recipient mice for 14 days. The fresh ovaries were only transplanted to the renal capsule of recipient mice for 14 days without freezing (Fig. 4).

About 74% of cultures in the 2 μl droplet vitrfication group and 69% in the 6 μl droplet vitrification group developed a thin transparent antral follicle (Fig. 1) during the 12 days culture period. There was no difference in the collection rates of cumulus oocyte complexes (COC) after 12 days of culture between the 6 μl droplet group and 2 μl droplet group (66% and 64%, respectively). The percentage of antral follicle formation and cumulus oocyte complexes collection in the 2 μl and 6 μl groups was less than that of the unfrozen preantral follicle control group (vs. 83% and 77%, respectively in control group) (Fig. 1).

When 14-day-old mice and newborn mice ovaries were vitrified by the droplet in metal surface method, use of a small volume of ovarian tissue slice and whole newborn ovaries of mice yielded a higher percentage of antral like cavity formation (61%, 54%, 37% respectively) and cumulus oocyte complexes collection (60%, 50%, 42% respectively) than that for 14-day old whole ovaries (p < 0.05) (Fig. 2). Higher percentages of granulosa cell outgrowth were also obtained in 14-day-old ovarian slices than in 14-day-old whole ovaries and whole newborn mice ovaries (94%, 73%, 61% respectively, p < 0.05) (Fig. 2).

### Oocytes maturation and in vitro fertilization of mouse preantral follicles derived from vitrified preantral follicles, vitrified ovarian slices, vitrified 2-week-old ovaries, and vitrified newborn mice ovaries

As shown in Fig 1 and Fig 2, no difference was found in the percentage of mataphase II oocytes collected and percentage of embryos developed to the 2-cell stage after fertilization of mouse preantral follicles derived from vitrified preantral follicles, vitrified ovarian tissue slices from 2-week-old mice, vitrified 2-week-old whole ovaries, and vitrified newborn mice ovaries. Nevertheless, the differences were all significant compared to that of the unfrozen control group (p < 0.05). No blastocyst stage embryos developed from 2-cell stage embryos was observed in freezing group of ovarian tissue slices, 2-week-old whole ovaries, and newborn ovaries groups. By comparison, 4.1%, 1%, 2% of 2-cell stage embryos developed to blastocyst stage embryos in unfrozen, 2 μl and 6 μl droplet vitrification groups of preantral follicles.

## Discussion

### Survival and development of vitrified-thawed preantral follicles

**Figure 5:**
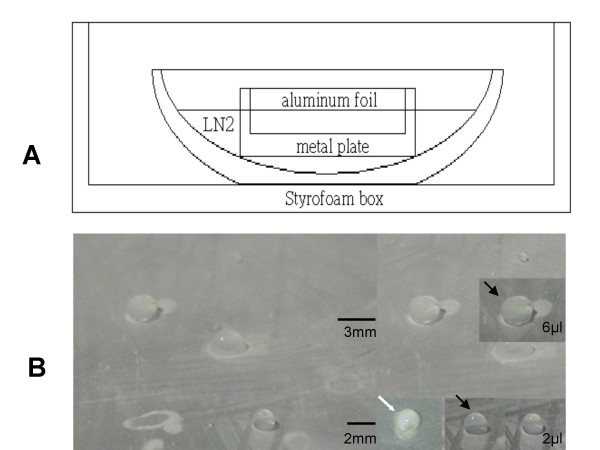
**The sketch of metal plate vitrification method and the formation of spherical transparent vitrified droplets**. The sketch of metal plate vitrification method shows a metal plate covered with aluminum foil cooled by partial immersion in LN2 contained in a 13 cm width vacuum steel bowl inside the Styrofoam box (A). The droplets, examples of 6 μl and 2 μl transparent vitrified droplets form a spherical shape at temperature of -196°C on the metal surface (distal bar = 3 mm, proximal bar = 2 mm) (B). Crystal transparency of the vitrified droplets (black arrow) is shown in different views. An obscure crystal droplet (white arrow) is shown which may occur after loading preantral follicles is characteristic of damage after thawing.

Although high survival rates were found for vitrified-thawed preantral follicles isolated from 14-day old unfrozen ovaries using the metal-plate surface method (-196°C) with 2 μl and 6 μl droplet groups, these rates were still lower than those obtained using unfrozen preantral follicles. Despite the expectation that the 2 μl or 6 μl sizes of these droplets would be sufficient to avoid this phenomenon of crystallization, extracellular crystallization of preantral follicle was still occasionally noted after vitrification and may have been an important factor responsible for intracellular damage in the 2 μl or 6 μl droplet groups which was associated with impacts on cell survival after thawing [26] (Fig. 5). Nevertheless, the survival rate in this study was 10% higher than that previously reported by dela Pena et al. [2] in vitrified preantral follicles using the stainless steel mesh method for cooling the droplet of vitrification solution [2] and other studies which used the slow freezing method with preantral follicles [38-40]. A similarly high cryosurvival rate (86%) was also obtained in vitrified-thawed bovine oocytes using the same solid (metal) surface method with 2 μl droplet [36]. The high cooling rate of metal surface vitrification method was achieved by using the combination of micro-drops and improved heat exchange via direct contact with a metal surface. The warming of preantral follicles or oocytes was equally rapid by directly dropping the vitrified samples into a warm solution. The small sample size of droplet also reduced the occurrence of preantral follicles cracking [36].

In this study, we found that increasing the droplet size from 2 μl to 6 μl did not affect the finding of vitrificating formation of transparent spherical droplets and similarly high survival rate was obtained in both groups. Hence in order to facilitate the handling of those specimens larger than the preantral follicles, we used the 6 μl volume only in the subsequent whole ovary and ovarian tissue slices vitrification experiments.

Using of droplet method for dropping the concentrated bovine spermatozoa onto the surface of dry ice or metal-plate (-79°C) was reported by Nagase in 1964 [41] and Hsu in 1968 [42] which they named as pellet form or capsule-pellet form semen. These studies obtained 5–10% higher conception rates using these techniques compared to convention freezing methods. Although Nagase's method [41] of freezing semen in pellet was not a vitrification method such as that described by Rall and Fahy's method for freezing the embryo [26], these studies demonstrated that the metal-plate provides a high cooling rate. When the concentrated semen solution was dropped onto the surface of dry ice or metal-plate, the concentrated cryoprotectant crystallized and formed a spherical pellet regardless of the solution volume used. When a high concentration of vitrification solution used for embryo cryoperservation was dropped onto the surface on dry metal-plate, the solution was vitrified when the volume was <2 μl. In present study, the method of spraying the liquid nitrogen was used to moisten the surface of metal-plate in the of 6 μl droplet groups. This was found to be an efficient method for avoiding the crystallization of the vitrification solution. Using our vitrification method for increasing the cooling rate between 20°C to -10°C, the "boiling off" phenomenon was disappeared. Papis et al. [31] as well as Landa and Tepla [30] vitrified bovine oocytes and 8-cell mouse embryo in 6 μl droplets, a minimal volume of vitrified sample, directly into a styrofoam box filled with liquid nitrogen. This method, in which the droplet plunges directly into liquid nitrogen, yielded promising results for development into blastocyts. Nevertheless, the authors noted that the "boiling off" phenomenon damaged the cell as a result of its plunge into liquid nitrogen.

In the present study, the vitrified preantral follicles were cultured according to the method of Cortvindt et al. [20] in a flat dish without supporting matrix. We obtained superior results in term of antral-like cavity formation, cumulus-oocyte complexes collection and MII collection than that from the report of dela Pena et al. [2], who cultured the thawed preantral follicles, vitrified and cryopreserved in a 0.25 ml straw, using the Transwell-COL membrane inserts method [19]. Our results for blastocyts development, however, were worse than that in their study. Differences in the culture system and in vitro fertilization system as well as the environment conditions of culture all may have affected the success rates of cultures in these studies.

### In vitro culture of preantral follicles

In present study, in vitro culture of the vitrified-thawed preantral follicles was used to assess developmental competence after thawing. There were two major methods reported to culture oocytes from primordial follicles. One is the follicle-culture system that embeds the follicles into collagen or agar gels or placed onto collagen-treated porous membrane to maintain close association between the oocyte and granulosa cells [2,19,20,43]. Another is organ-follicle-culture system using a two-step strategy for oocyte development from primordial follicles. In this method, intact ovaries from newborn mice were organ-cultured for 8-days, and oocyte-granulosa cell complexes isolated from them were cultured on Millipore – PC membrane inserts for 14 days [21].

Cortvindt et al. [20] had simplified follicle-culture system. The follicles were cultured singly in 20 μl droplets under oil in medium supplemented with recombinant follicle stimulating hormone (r-FSH). With the application of this culture method, fertilizable oocytes can also be obtained by culturing the follicles on flat tissue culture dishes [44]. Under these conditions, adhering theca cells function as "substrate" for subsequent granulosa cells proliferation and outgrowth through the basement membrane. The loss of follicle spherical aspect in these cultures does not interfere with antral cavity formation and the acquisition of full oocyte developmental potential [45].

In the present study, we adopted the method devised by Cortvindt et al. [20] using culture in inexpensive flat dishes to determine the developmental competence of vitrified-thawed preantral follicles from 14-day old mouse ovaries. The results indicated the development of granulosa cell outgrowth, antral-like cavity formation, cumulus-oocyte complexes (COC) collected, MII oocytes retrieved, and 2-cell embryos and blastocysts developing after in vitro fertilization were not significantly different between those in 2 μl and 6 μl groups. But these rates were lower than the results obtained in unfrozen control groups (Fig. 1).

### Survival and development of preantral follicles isolated from vitrified-thawed ovaries

In this report, although the survival rate after thawing was not different between the ovarian tissue slices and intact ovary groups, the granulosa outgrowth and the antral-like cavity formation were significantly different (p < 0.05) in the in vitro growth cultures. No significant difference was found between ovarian tissue and intact ovary in COC collected, MII collected, 2-cell stage and 8–16 cell stage. These results may suggest that the equilibration time of the vitrification solution or the thawing solution might not have been sufficient in this protocol for whole ovary vitrification.

A similar study was previously conducted by Liu and He in 2003 [22], who cryopreseved 14-day-old mouse ovarian tissue using the slow freezing method and isolated the preantral follicles. After in vitro growth and in vitro maturation of follicles, they found no significant differences in IVM outcomes between the fresh and frozen experimental groups. They also noted that cryopreservation appeared to induce the expression of heat shock proteins, DNA-damage-inducible protein 45 and death-related apoptosis.

Zhang et al. 2004 [46] studied the feasibility of intact mouse ovaries cryopreservation by vitrification. In their study, intact mouse ovaries were cryopreserved using the vitrification method and slow freezing method. They reported that no significant difference in the primordial follicles survival rate and the apoptosis rates was found between the two cryopreservation groups. After the freezing-thawing procedure, subcellular structure was well preserved in both freezing groups with similar ultrastructurall changes.

### Development of preantral follicles isolated from vitrified-transplanted newborn ovaries

In our present study we demonstrated that newborn mouse ovaries cryopreserved using the metal-plate vitrification in 6 μl droplet resulted in a high survival rate of preantral follicles. These preantral follicles from vitrified-thawed-transplanted newborn mice ovaries were capable of developing antral-like cavity formation, responded to hormonal stimulation, and yielded oocytes that could be fertilized, with development at least to the 8–16 cells stage. Although the results were less successful than with unfrozen day-14 preantral follicles and preantral follicles isolated from vitrified day-14 ovarian tissue, they were still better than that obtained with vitrified whole ovaries from 14-day mice (Fig. 2). Similar research was conducted by Liu et al. in fresh [47] and frozen [3] newborn mice ovaries and they resulted in live offspring by in vitro fertilization of oocytes from cryopreserved primordial mouse follicles after sequential in vivo transplantation and in vitro maturation [3]. However, they froze the newborn mice ovaries by slow freezing method in a 0.25 ml straw. By contrast, in the present study, we used the pre-pubescent male mice as recipients for transplantation. Nevertheless, a comparable result was obtained in the survival rate of preantral follicles, antral-like cavity formation and oocyte maturation.

## Conclusion

This study demonstrated that the two-week-old preantal follicles, 2-week-old whole ovaries, ovarian tissues and newborn mice whole ovaries preserved using this vitrification protocol by dropping the droplet onto the surface of metal surface yielded high rate of follicle survival after thaw, COCs collection, MII oocytes formation, and comparable rate of two cell embryos development after subsequent transplantation, in vitro growth, in vitro maturation and in vitro fertilization and culture. This method of vitrification is simple and requires only fairly simple equipment such as a metal plate and a vacuum bowl and a styrofoam box. The metal plate can be placed in a liquid nitrogen tank for storage at -196°C. Only a small mount of liquid nitrogen is needed to maintain the metal plate at this temperature during cryopreservation. It is thus an economically feasible method for use in varies applications. However, factors affecting the developmental competence including the overall environmental conditions must be controlled.

## Methods

### Animals

The female and male C57BL/6 mice used in this study were housed and bred in the animal house of Kuo General Hospital. Males that provided sperm were at least 3 months old and were caged individually since weaning. All mice were housed under a 14-h light, 10-h dark cycle at 25 ± 1°C. One-day-old females were used for collection of newborn mouse ovaries. Two-week-old female were used for isolation of preantral follicles from ovaries prior to freezing or after freezing. Three- to four-week-old male mice were used as recipients for newborn ovarian transplantation. Newborn intact ovaries collected from 1-day-old female mice were cryopreserved prior to transplantation. Two weeks after ovarian transplantation, recruited preantral follicles were isolated from grafted ovaries. The females were killed by cervical dislocation. Ovaries were collected in Leibovitz's L-15 medium (L-15, Gibco BRL. 21083) supplemented with 10% fetal bovine serum (FBS, Sigma F-4135), 75 mg/l penicillin G (Sigma P-3032) and 50 mg/l streptomycin sulfate (Sigma S-9137) at 37°C.

### Vitrification and thawing of preantral follicles and ovaries

In order to simplify the procedure of solid surface vitrification and to adequately accommodate the volume of the intact ovaries being frozen, we modified the vitrification procedure described by Dinnyes et al. [34]. Briefly, groups of 5 to10 preantral follicles or 2–4 ovaries were exposed to 4% ethylene glycol (EG) in DPBS + 10% FBS for 15 min. The preantral follicles or ovaries were rinsed three times in a vitrification solution composed of 6 M ethylene glycol and 0.4 M trehalose in DPBS + 10% FBS equilibrated in a room temperature bath for 20–30 seconds for preantral follicle vitrification and in an ice bath for 5 min for ovaries vitrification. After equilibration, the preantral follicles or ovaries were dropped directly onto the surface of a metal plate (anodized aluminium) covered with aluminum foil and cooled to around -150 to -180°C by partial immersion in LN_2_. The droplets, containing vitrification solution and one to three preantral follicles, in size of 2 μl, were instantaneously vitrified into a transparent spherical droplet. Droplets, containing one ovary, one ovarian tissue slice (bisection the ovary twice with bio-cut blade or fine forceop to the size of 0.8 × 0.3 mm), or five preantral follicles, in size of 6 μl were dropped on the surface of aluminum foil that had been sprayed with LN2 before use to avoid the droplets taking a dome shaped form or fried egg-like form and adherence of the vitrification solution to the surface of foil. The droplets were moved with liquid nitrogen cooled forceps into 1-ml cryovials and then preserved in LN2 for long-term storage. They were thawed by dropping into a thaw solution composed of 0.3 M trehalose in DPBS and 10% FBS at 37°C for 5–10 min, and washed with DPBS three times. The vitrified-thawed preantral follicles were collected for IVG culture. The vitrified-thawed 2-week-old ovaries from 2-week-old mice were collected and used for isolation of the preantral follicles. To avoid the large amount of liquid nitrogen consumption as the metal plate was cooled, we optimized the size of metal plate to 10 × 6 × 3 cm^3 ^and stored it in liquid nitrogen tank for maintenance the temperature of metal plate in -196°C at any time for use. A 13 cm width vacuum steel bowl placed inside the Styrofoam box was used as a container for the liquid nitrogen and metal plate (Fig. 5).

### Transplantation of vitrified-thawed newborn ovaries

**Figure 6:**
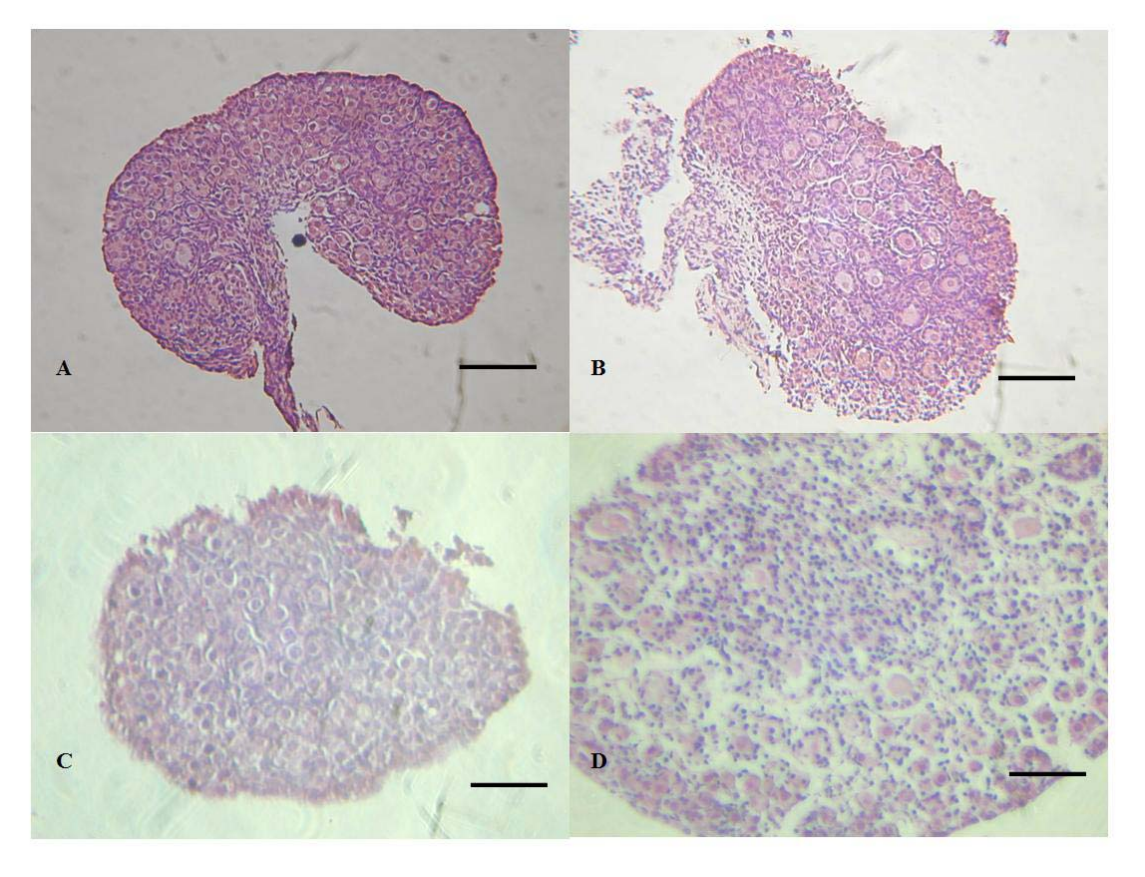
**Histological section of frozen-thawed newborn mouse ovary**. Histological section illustrating the appearance of a fresh ovary isolated from a newborn mouse (A) (bar = 100 um). Histological section of frozen-thawed new born ovary with metal surface vitrifiation before transplantation (B) (bar = 100 um). Dead (C) and survival ovary (D) after transplantation under the renal capsule for 14 days (bar = 100 um).

The recipient male mice were anesthetized with i.p. injection of 0.017 ml/g body weight of avertin. A stock of 100% avertin was prepared by mixing 10 g of 2,2,2-tribromoethyl alcohol (Fluka 90710) with 10 ml of tetra-amyl alcohol (Fluka 66000), which was diluted to 2.5% in water or saline for use. The transplantation procedure used was a modification of the method of Gosden et al. [48]. Briefly, the left kidney was exteriorized through a dorsal-horizontal incision. A small hole was torn in the kidney capsule using fine microsurgical forceps. Two whole vitrified-thawed newborn mouse ovaries were inserted through the small hole underneath the renal capsule of each recipient mouse. The recipient male mice were injected with 5 IU r-FSH (Serono, Aubone, Switzerland) every other day during the 14 days post-transplantation period (Fig. 6).

### Isolation of preantral follicles

Preantral follicles were mechanically isolated from the mouse ovaries using a 27^1^/_2_G needle attached to a 1 ml syringe in L-15 + 10% FBS at 37°C. Morphologically normal preantral follicles with two to three layers of granulosa cells and centrally located spherical oocytes were used in the experiments.

### In vitro growth and in vitro maturation of preantral follicles

**Figure 7:**
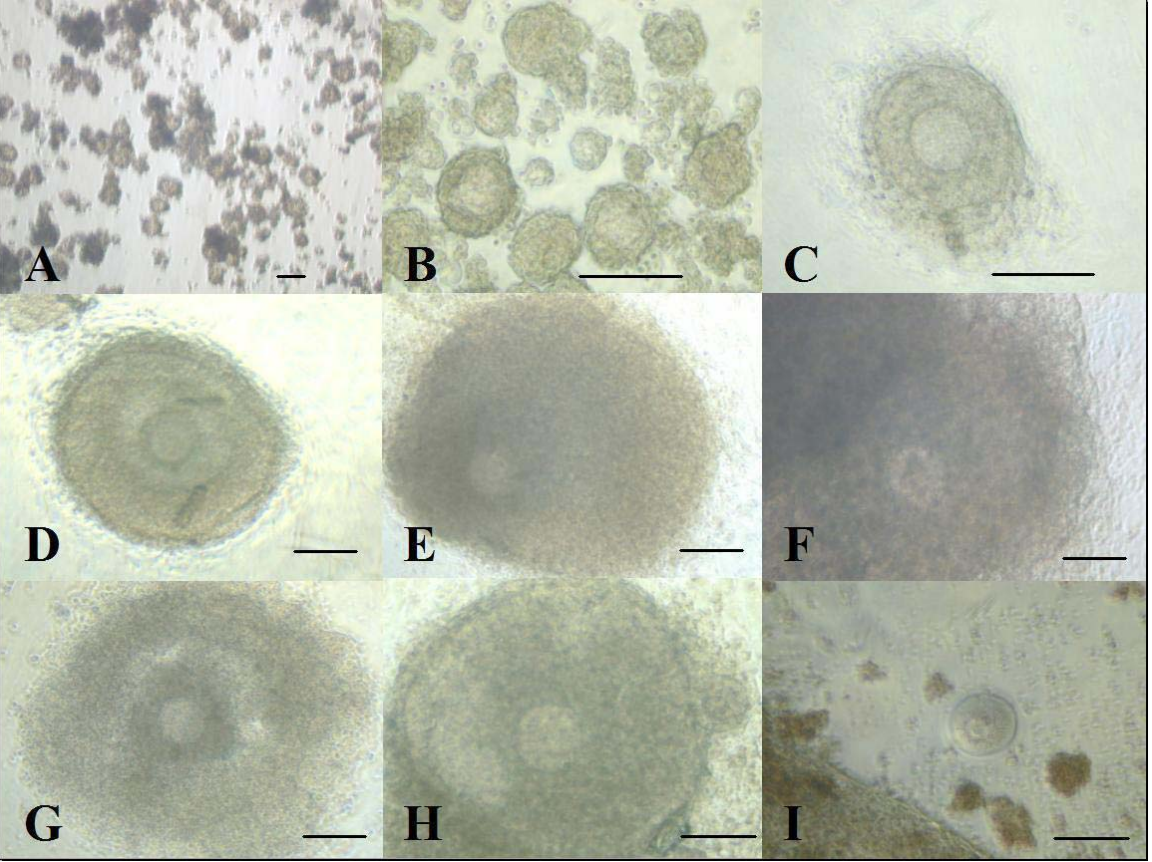
**Granulosa outgrowth and antral-like cavity formation of cultured vitrified-thawed preantral follicles**. Vitrified-thawed preantral follicles (from 14-day old mouse) cultured in vitro for 12 days: Vitrified-thawed preantral follicles (A) had just been isolated, cultured for 3 days (B), cultured for 6 days with granulosa outgrowth (C) (bar = 100 um). Antral-like cavity formation (D-H) (bar = 100 um) with different antral stages and ovulated oocyte (I) with nearby granulosa cells were observed.

The washed preantral follicles were cultured in the IVG medium described by Cortvrindt et al. (1996 a) [20] with some modification: The culture medium was composed of α-minimal essential medium (α-MEM) (Gibco 32561) supplemented with 5% heat-inactivated FBS (Life Technology), 1× insulin (1.0 g/L)-transferrin (0.55 g/L)-selenium (0.67 mg/L) (Gibco 41400-045), and 100 mIU/ml recombinant follicle stimulating hormone (r-FSH, LH-free) (Serono, Aubone, Switzerland). Culture dishes (35 mm Petri dishes; Falcon, Becton Dickinson) contained 10 × 10 μl culture droplets and 2 × 10 μl washing droplets, covered with 2 ml of washed mineral oil (Sigma, M-8410). Selected early preantral mouse follicles were washed in the washing droplets and placed one by one in the culture droplets. On day 2 of culture, 10 μl of medium was added to each droplet and thereafter half of the medium was refreshed every other day. Follicles were cultured in an incubator at 37°C, 100% humidity and 5% CO_2 _in air. The diameter of the basal membrane and granulosa cell outgrowth of preantral follicles was measured every other day during the culture period (Fig. 7).

On day 12 of culture, the cumulus-oocyte complexes(COCs) were collected and the morphologically normal cumulus-oocyte complexes were transferred to in vitro maturation culture medium and incubated 14–16 h at 37°C in humidified atmosphere of 5% CO_2 _in air. The in vitro maturation culture medium was composed of α-minimal essential medium (α-MEM) (Gibco 32561) supplemented with 0.1% serum replacement 1 (Sigma S-0638), insulin(10 μg/ml)-transferrin(5.5 μg/ml)-selenium(0.65 ng/ml) (Gibco 41400-045), 2.5 IU/ml human chorionic gonadotrophin (Serono, Aubonne, Switzerland) and 10 ng/ml human epidermal growth factor (Sigma E-9644). At the end of in vitro maturation culture, oocyte maturation status was classified as GV when the germinal vesicle (GV) was present, as GVBD (GV breakdown) when the GV had broken down, and as metaphae II when the first polar body had been extruded (Fig. 7).

### In vitro fertilization and culture

After in vitro maturation culture, oocytes surrounded with cumulus cells were inseminated in vitro using the procedure described by Liu et al. [3]. Briefly, the caudae epididymis were removed from 3–6 month old F1 males (C57BL/6J × C3H) and placed into 1 ml drops of potassium simplex optimized medium (KSOM) supplemented with 0.4% BSA under minimal oil (Sigma, M-8410) in culture dish. Epididymis contents were squeezed out and were placed in incubator for 20 min to allow the sperm to disperse. Ten μl of sperm suspension was added to 90 μl drops of KSOM+BSA in the fertilization dishes. Capacitation was allowed to proceed for 45–60 min at 37°C in the incubator. After capacitation, COCs were transferred to the 100 μl fertilization droplets (10 μl sperm suspension + 90 μl KSOM + BSA). Fertilization was allowed to proceed for 4 h at 37°C in a 5% CO2 incubator. At the end of culture, the cumulus cells and attached sperm were removed from the oocytes by drawing the oocytes in and out of a fine-drawn pipette. After five times rinsing, about 10 inseminated oocytes were incubated in 20 μl droplets of KSOM+BSA in culture dishes. Fertilized oocytes were cultured in 20 μl drops of KSOM+BSA under 2 ml mineral oil in culture dishes for approximately 72 h without changing the medium. Fertilization was scored as the percentage of cleavage to the 2 cell stage embryos on day 2.

### Statistical analysis

Data were presentated as mean percentages of at least five independent experimental replicates; variation between experiments is illustrated using the standard error of the mean. For evaluation of the differences between two groups, data for the replicates were pooled and subjected to chi-square analysis; a value of P < 0.05 was considered significant. The mean percentages did not vary from the pooled percentages by more than 5%.

## Abbreviations

COCs: Cumulus-oocyte complexes; IVG: In vitro growth; IVM: In vitro maturation; EG: Ethylene glycol; DPBS: Dulbecco's phosphate buffer solution; KSOM: Potassium simplex optimized medium.

## Authors' contributions

TCL conceived of the study, participated in its design, and drafted the manuscript. JMY participated in the design of the study and performed the statistical analysis. KBG and KHH performed the follicle and embryo culture. TCL and KBG carried out the micromanipulation and microscopic observation. TCK helped to plan and coordinate the studies. TTH supervised and interpreted the studies and helped manuscript preparation.
